# Rationale for Prolonged Glucocorticoid Use in Pediatric ARDS: What the Adults Can Teach Us

**DOI:** 10.3389/fped.2016.00058

**Published:** 2016-06-14

**Authors:** Andreas Schwingshackl, Gianfranco Umberto Meduri

**Affiliations:** ^1^Department of Pediatrics, Division of Critical Care Medicine, Mattel Children’s Hospital at UCLA, Los Angeles, CA, USA; ^2^Departments of Medicine, Division of Pulmonary, Critical Care, and Sleep Medicine, Memphis Veterans Affairs Medical Center, Memphis, TN, USA

**Keywords:** acute respiratory distress syndrome, glucocorticoid treatment, mechanical ventilation, survival

## Abstract

Based on molecular mechanisms and physiologic data, a strong association has been established between dysregulated systemic inflammation and progression of acute respiratory distress syndrome (ARDS). In ARDS patients, glucocorticoid receptor-mediated downregulation of systemic inflammation is essential to restore homeostasis, decrease morbidity and improve survival and can be significantly enhanced with prolonged low-to-moderate dose glucocorticoid treatment. A large body of evidence supports a strong association between prolonged glucocorticoid treatment-induced downregulation of the inflammatory response and improvement in pulmonary and extrapulmonary physiology. The balance of the available data from eight controlled trials (*n* = 622) provides consistent strong level of evidence for improving patient-centered outcomes and hospital survival. The sizable increase in mechanical ventilation-free days (weighted mean difference, 6.48 days; CI 95% 2.57–10.38, *p* < 0.0001) and intensive care unit-free days (weighted mean difference, 7.7 days; 95% CI, 3.13–12.20, *p* < 0.0001) by day 28 is superior to any investigated intervention in ARDS. For treatment initiated before day 14 of ARDS, the increased in hospital survival (70 vs. 52%, OR 2.41, CI 95% 1.50–3.87, *p* = 0.0003) translates into a number needed to treat to save one life of 5.5. Importantly, prolonged glucocorticoid treatment is not associated with increased risk for nosocomial infections (22 vs. 27%, OR 0.61, CI 95% 0.35–1.04, *p* = 0.07). Treatment decisions involve a tradeoff between benefits and risks, as well as costs. This low-cost, highly effective therapy is familiar to every physician and has a low risk profile when secondary prevention measures are implemented.

## Physiologic Considerations for Prolonged Glucocorticoid Therapy in ARDS

**Figure 1:**
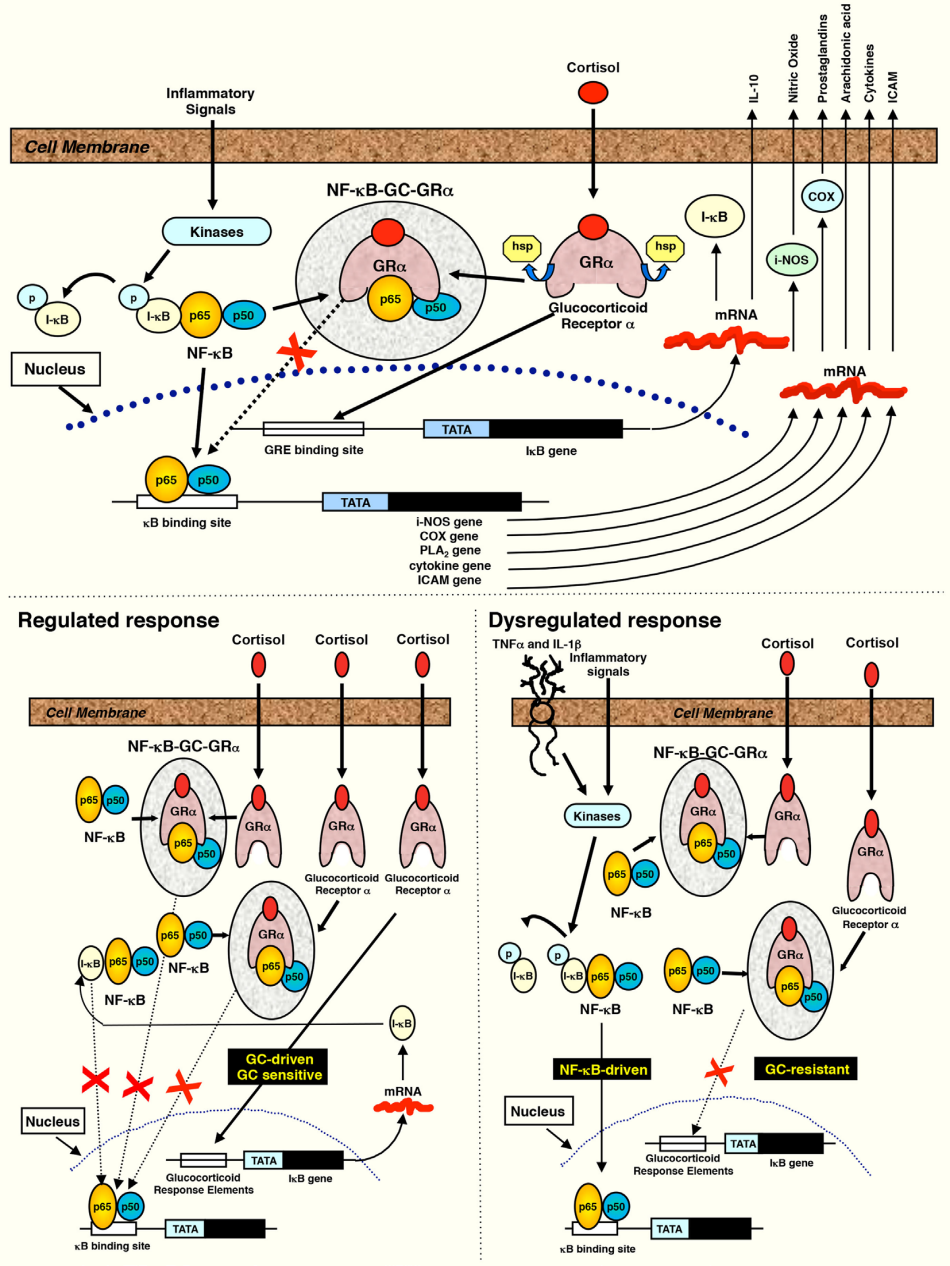
**Interaction between NF-κB and the activated glucocorticoid receptor**. Reproduced from Ref. (3) with permission from S. Karger AG, Medical and Scientific Publishers (Allschwilerstrasse 10, 4009 Basel, Switzerland). When cells are stimulated by inflammatory signals, specific kinases phosphorylate the inhibitory protein IκB and cause its rapid degradation. The activated form of NF-κB then moves to the nucleus initiating the transcription of mRNA of inflammatory cytokines, chemokines, cell adhesion molecules, and inflammation-associated enzymes (cyclooxygenase, phospholipase A2, and inducible nitric oxide). Cortisol or exogenous glucocorticoids freely cross into the cytoplasm and bind to their specific glucocorticoid receptors (GRα) to form the activated receptor (GC-GRα). GC-GRα complexes may influence NF-κB activity in five major ways: (1) physically interacting with the p65 subunit with formation of an inactive (GC-GRα/NF-κB) complex (4, 5), (2) inducing the synthesis of the inhibitory protein IκBα *via* interaction with glucocorticoid-responsive element DNA in the promoter of the IκB gene (5–7), (3) blocking degradation of IκBα *via* enhanced synthesis of IL-10 (8–10), (4) impairing TNF-α-induced degradation of IκBα (11, 12), and (5) competing for limited amounts of GR coactivators, such as CREB-binding protein (CBP) and steroid receptor coactivator-1 (SRC-1) (13). IκBα, in addition to holding NF-κB in an inactive cytoplasmic state (5–7), also translocates into the nucleus, where it binds activated NF-κB complexes to induce their export to the cytoplasm (4). GC-GRα may also decrease the stability of mRNA of several inflammatory cytokines and other molecules (14). Products of the genes that are stimulated by NF-κB activate this transcription factor. Thus, both TNF-α and IL-1β activate and are activated by NF-κB, by forming a positive regulatory loop that amplifies and perpetuates inflammation (15). *Regulated response*: GC-GRα activation sufficient to maintain NF-κB levels in homeostasis and achieve a reduction in transcription of inflammatory mediators over time is shown. In ARDS improvers, both GC-GRα binding to NF-κB and nuclear GC-GRα binding increased significantly over time, indicating an excess activation of GC-GRα compared to NF-κB (GC-GRα-driven response). *Dysregulated response*: an excess of NF-κB activation is shown, leading to protracted transcription of inflammatory mediators over time. In ARDS non-improvers, GC-GRα binding to NF-κB was modestly increased while nuclear NF-κB binding increased substantially over time and nuclear GC-GRα and cytoplasmic IκBα levels declined (NF-κB-driven response).

Acute respiratory distress syndrome (ARDS) is a secondary disease that follows – usually within 6–48 h – a primary disease of multifactorial etiology (most frequently pneumonia and extrapulmonary sepsis) associated with severe systemic inflammation. Inflammatory mediators released into the systemic circulation (systemic inflammation) from the site of infection reach the broad pulmonary capillary surface producing severe and diffuse inflammatory exudate of the pulmonary lobules resulting in hypoxemic respiratory failure (1, 2). In sepsis and ARDS, systemic inflammation is activated by the nuclear factor-κB (NF-κB) signaling system and downregulated by the activated glucocorticoid receptor-α (GRα) (1) (Figure 1). In these patients, inadequate (endogenous glucocorticoid activated) GRα-mediated downregulation of pro-inflammatory NF-κB in circulating and tissue cells leads to higher initial levels and persistent elevation over time in plasma and bronchoalveolar lavage (BAL) markers of inflammation, hemostasis, and tissue repair (1).

In ARDS, “resolution of inflammation and restoration of tissue homeostasis is an active, continuous, and coordinated process (switch from production of pro-inflammatory toward pro-resolving molecules)” (16) – characterized by non-phlogistic elimination of granulocytes by apoptosis, restoration of the alveolar-capillary membrane’s integrity, resorption of edema, increased surfactant production, decreased clotting, and fibroproliferation with resolution of intra-alveolar and interstitial granulation tissue (1, 2) – that can be accelerated by prolonged glucocorticoid treatment (17). There is a strong scientific foundation in support of prolonged methylprednisolone treatment in ARDS, with evidence showing that this treatment is directed at the core pathogenic mechanism of ARDS and as a result positively affects all “layers” – biology, histology, and physiology – of the disease process (18).

Importantly, in ARDS the inadequate GRα-mediated downregulation of pro-inflammatory transcription factor NF-κB in circulating and tissue cells leads to persistent elevation over time (>4 weeks) in plasma and BAL markers of inflammation, hemostasis, and tissue repair (maladaptive repair) (1). In experimental sepsis, inhibition of endogenous glucocorticoid synthesis aggravates acute lung injury, while glucocorticoid treatment was associated with protection (19). Experimental ARDS is associated with a significant reduction in lung tissue GRα expression (20–22) and increase in GRβ mRNA (21), leading to decreased GRα nuclear translocation (21). In these experiments, low-dose glucocorticoid treatment – contrary to placebo – restored GRα number and function, leading to resolution of pulmonary inflammation (22, 23). Moreover, alveolar macrophages extracted from patients with established ARDS have decreased 11β-HSD oxo-reductase activity with decreased conversion of cortisone to cortisol (24).

**Figure 2:**
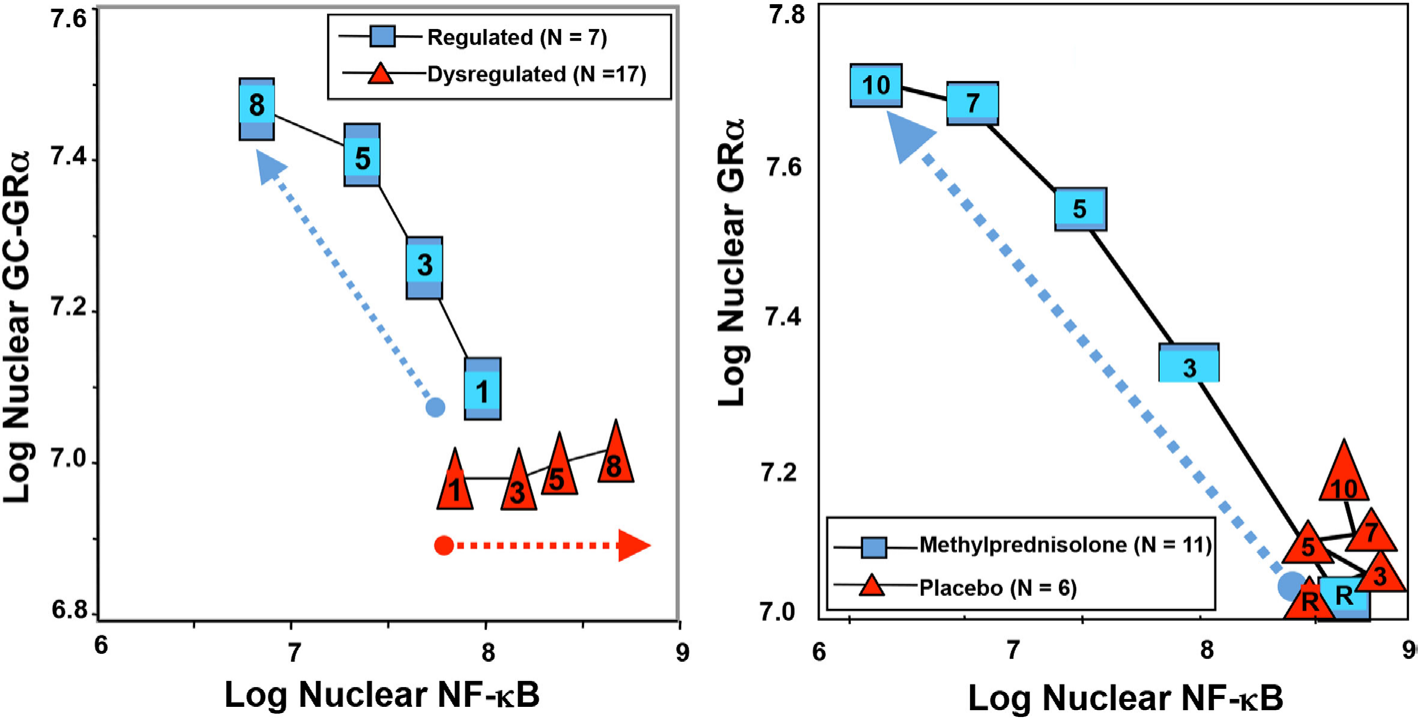
**Longitudinal relation on natural logarithmic scales between mean levels of nuclear NF-κB and nuclear GRα: resolving vs. unresolving ARDS (left) and after randomization to methylprednisolone vs. placebo (right)**. Data shown are from Ref. (3, 25). *Left*: plasma samples from patients with sustained elevation in cytokine levels over time (triangles) elicited only a modest longitudinal increase in GC-GRα-mediated activity (*P* = 0.04) and a progressive significant (*P* = 0.0001) increase in NF-κB nuclear binding over time (dysregulated, NF-κB-driven response). In contrast, in patients with regulated inflammation (squares), an inverse relation was observed between these two transcription factors, with the longitudinal direction of the interaction shifting to the left (decreased NF-κB) and upward (increased GC-GRα). The first interaction is defined as NF-κB-driven response (progressive increase in NF-κB–DNA binding and transcription of TNF-α and IL-1β) and the second interaction as GRα-driven response (progressive increase in GRα-DNA binding and transcription of IL-10 and repression of TNF-α and IL-1β). *Right*: longitudinal relation on natural logarithmic scales between mean levels of nuclear NF-κB and nuclear GRα observed by exposing naive PBL to plasma samples collected at randomization (rand) and after 3, 5, 7, and 10 days in the methylprednisolone (squares) and placebo (triangles) groups. With methylprednisolone, contrary to placebo, the intracellular relation between the NF-κB and GRα signaling pathways changed from an initial NF-κB-driven and GR-resistant state to a GRα-driven and GR-sensitive one. It is important to compare the two figures to appreciate how methylprednisolone supplementation restored the equilibrium between activation and suppression of inflammation that is distinctive of a regulated inflammatory response.

In an *ex vivo* study, prolonged methylprednisolone treatment – contrary to placebo – was associated with upregulation in GRα number and nuclear translocation with reduction in NF-κB–DNA binding and transcription of inflammatory cytokines (25) (Figure 2). ARDS patients randomized to prolonged methylprednisolone treatment, contrary to placebo, demonstrated a sustained reduction in plasma and/or BAL levels of tumor necrosis factor-α (TNF-α), interleukin (IL)-1β, IL-6 (1), IL-8, soluble intercellular adhesion molecule-1 (1), procollagen aminoterminal propeptide type I and III (1), indices of alveolar-capillary membrane permeability (BAL albumin, total protein, and percentage neutrophils) (26), and an increase in IL-10 (1), protein C (27), and surfactant (28).

## Prolonged Glucocorticoid Treatment in ARDS is Associated with Improved ARDS Outcomes: Lessons from Adult ARDS Studies

The updated literature on prolonged glucocorticoid treatment in adult patients with ARDS includes eight published randomized controlled trials (RCTs): four investigated methylprednisolone treatment initiated in early (<72 h; *n* = 118) (29, 30) or late ARDS (day 5–28; *n* = 204) (31, 32), and four investigated 7 days of hydrocortisone treatment in early ARDS (*n* = 297) (33–36). Additional publications include, one RCT recently presented in abstract form (*n* = 197) (37), seven cohort studies with historical or concurrent control (38–44), and eight observational reports (45–52).

As shown in Table 1, glucocorticoid administration, in comparison to placebo, was associated with a significant downregulation of systemic inflammation [reduction in inflammatory cytokines (29, 31, 32, 34), protein C (27, 30), and/or C-reactive protein levels (29–31, 33, 35)]. This biological response was associated with significant improvement in PaO_2_:FiO_2_ (29–36), and reduction in duration of mechanical ventilation (29–33, 35), and intensive care unit (ICU)-length of stay (1, 29, 30, 32, 33, 36). These consistently reproducible findings (29–36) “provide additional support for a causal relationship between reduction in systemic inflammation and resolution of ARDS that is further reinforced by experimental (53–56) and clinical (31, 38, 45, 46, 53, 57) data, showing that rebound inflammation following early removal of glucocorticoid treatment leads to recrudescence of ARDS that improves with reinstitution of glucocorticoid therapy” (58).

**Table 1:** **Randomized trials investigating prolonged glucocorticoid treatment in early and late ARDS: level of significant difference (*p* value) – in comparison to control – for anti-inflammatory therapeutic effect and clinical–physiological parameters of disease resolution**.

Study (*n* = 619)	Reduction in markers of systemic inflammation^a^	Improvement in PaO_2_:FiO_2_	Reduction in duration of assisted breathing	Reduction in ICU length of stay
Meduri et al. (31) (*n* = 24)	0.0001	0.001	0.001	0.005
Confalonieri et al. (33) (*n* = 34)^b^	0.01	0.001	0.007	0.001
Annane et al. (34) (*n* = 177)	0.01	0.001	No^c^^,^^d^	Not reported
Steinberg et al. (32) (*n* = 180)	0.001	0.02	0.001^c^	0.02^c^
Meduri et al. (29) (*n* = 91)	0.0001	0.006	0.002	0.007
Sabry and Omar (35) (*n* = 60)^b^	0.0001	0.001	0.01	Not reported
Liu et al.(36) (*n* = 26)	Not reported	Yes^||^	Not reported	Yes^||^
Rezk and Ibrahim (30) (*n* = 27)	0.0001	0.001	0.001	0.001
Proportion of studies with significant difference	100%	100%	87%	100%

*ICU, intensive care unit*.

*^a^Glucocorticoid administration was associated with a significant downregulation of systemic inflammation [reduction in inflammatory cytokines (29, 31, 32, 34), protein C (27, 30) and/or C-reactive protein levels (29–31, 33, 35)]*.

*^b^The data from Confalonieri et al. (33) and Sabry at al. (35) are limited to patients on assisted breathing*.

*^c^In the randomized trials by Annane et al. (34) and Steinberg et al. (32), data were reported only as ventilator-free (32, 34) and ICU-free days to day 28 (32)*.

*^d^In the randomized trials by Annane (34), a significant (*p* = 0.006) reduction in ventilator-free days was observed only in the larger subgroup (*N* = 129) of non-responders to a short corticotropin test*.

*^||^*p* value not available*.

**Figure 3:**
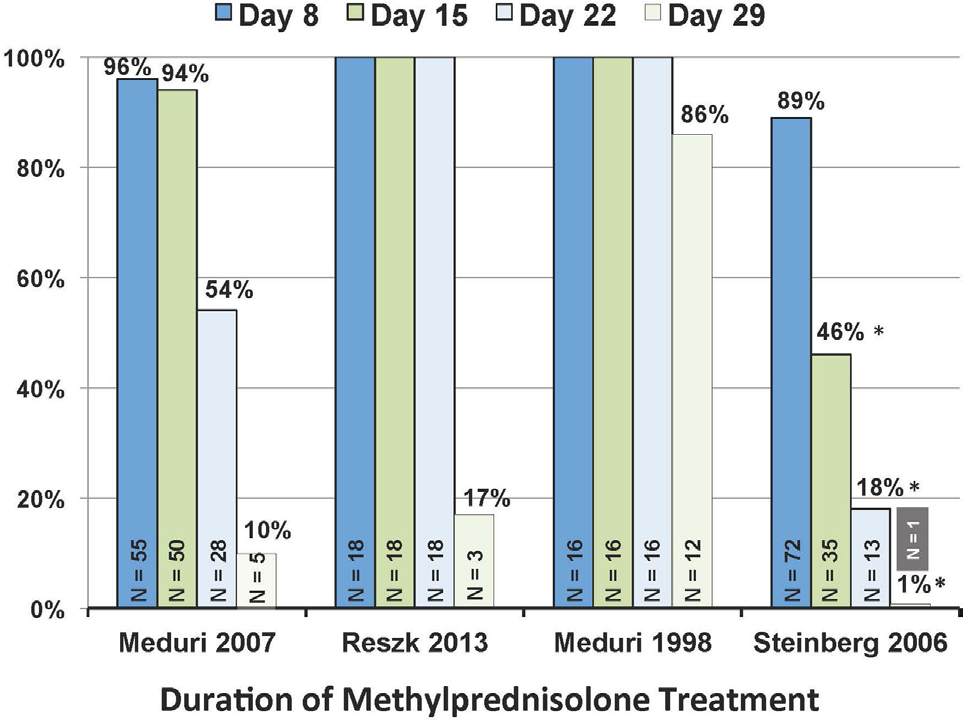
**Patients, in each trial, alive on study day 8, 15, 22, and 29 that received at least 7, 14, 21, and 28 days of methylprednisolone treatment**. *N* value within each column reports patients alive on the specified day. *P < 0.001 in comparison to the other three trials. Reproduced with permission (58).

In a recent systematic review (58), we evaluated the effectiveness of prolonged glucocorticoid treatment in ARDS with two sets of intention-to-treat analyses: a primary analysis of individual patients data (IPD) of the four methylprednisolone RCTs (*n* = 322) (29–32) and a trial-level meta-analysis incorporating methylprednisolone and hydrocortisone RCTs (*n* = 297) (33–36). In the primary IPD analysis, definitions were standardized to derive outcomes of four RCTs investigating methylprednisolone treatment initiated in early (<72 h; *n* = 118) (29, 30) and late (after 5–7 days; *n* = 204) (1, 32) ARDS. In the early (29, 30) and late ARDS trials (31, 32), the initial daily methylprednisolone dosage was 1 and 2 mg/kg/day and duration of treatment extended up to 25 and 32 days, respectively. Since these RCTs differed in timing of treatment initiation [early (29, 30) vs. late (31, 32)] and speed of drug tapering [slow (29–31) vs. rapid (32)] after achieving initial unassisted breathing (UAB), the IPD analysis evaluated the impact of these treatment characteristics on outcomes. As shown in Table 2, glucocorticoid administration, in comparison to placebo, was associated with accelerated resolution of ARDS improving a broad spectrum of interrelated clinical outcomes and decreasing hospital mortality and health-care utilization. “By day 28, the methylprednisolone group achieved initial UAB earlier (12.4 ± 0.61 vs. 19.8 ± 0.78 days; HR 2.59, 95% CI 1.95–3.43, *p* < 0.001; Figure 3) and the effect was similar after adjusting for pre-specified covariates. Incorporating data past day 28, the methylprednisolone group also had shorter duration of initial AB (12.9 ± 13.4 vs. 23.0 ± 13.9 days, mean difference −10.1, 95% −13.12 to 7.08, *p* < 0.001; high certainty) (58). In the trial-level meta-analysis, glucocorticoid treatment was associated with a sizable and significant increase in MV-free days (13.3 vs. 7.6 days; mean difference 5.76, 95% CI 3.76–11.52; high certainty), and in ICU-free days (10.8 vs. 6.4 days; mean difference 4.45, 95% CI 2.64–6.26; moderate certainty)” (58).

**Table 2:** **Effects of prolonged methylprednisolone treatment based on individual patient data from four randomized clinical trials of acute respiratory distress syndrome**.

Outcome variables	Methylprednisolone (*N* = 186)	Placebo (*N* = 136)	Odds ratio (95% CI) or LS means difference (95% CI)
Died before achieving initial UAB by day 28 – *n* (%)	23 (12)	39 (29)	0.35 (0.20, 0.63), *p* < 0.001
Alive on day 28 on initial AB with no UAB – *n* (%)	14 (8)	29 (21)	0.35 (0.173, 0.69), *p* = 0.003
Achieved initial UAB by day 28 – *n* (%)	149 (80)	68 (50%)	3.77 (2.29, 6.23), *p* < 0.001
Mechanical ventilation-free days by day 28 – mean ± SE	13.3 ± 11.8	7.6 ± 5.7	5.76 (3.76, 11.52), *p* < 0.001
Duration of initial AB including data past day 28 – mean ± SD	12.9 ± 13.4	23.0 ± 13.9	−10.10 (−13.12, −7.08), *p* < 0.001
Intensive care unit-free days up to day 28 – mean ± SE	10.8 ± 0.71	6.4 ± 0.85	4.45 (2.64, 6.26), *p* < 0.001
Hospital-free days up to day 28 – mean ± SE	7.0 ± 0.57	3.82 ± 0.68	3.19 (1.74, 4.64), *p* < 0.001
Hospital mortality – *n* (%)	37 (20)	45 (33)	0.48 (0.29, 0.81), *p* = 0.006
Hospital mortality for those randomized before ARDS day 14 (*n* = 272) – *n* (%)	32 (20)	43 (39)	0.39 (0.22, 0.67), *p* < 0.001

*Reproduced with permission from Ref. (58)*.

**Figure 4:**
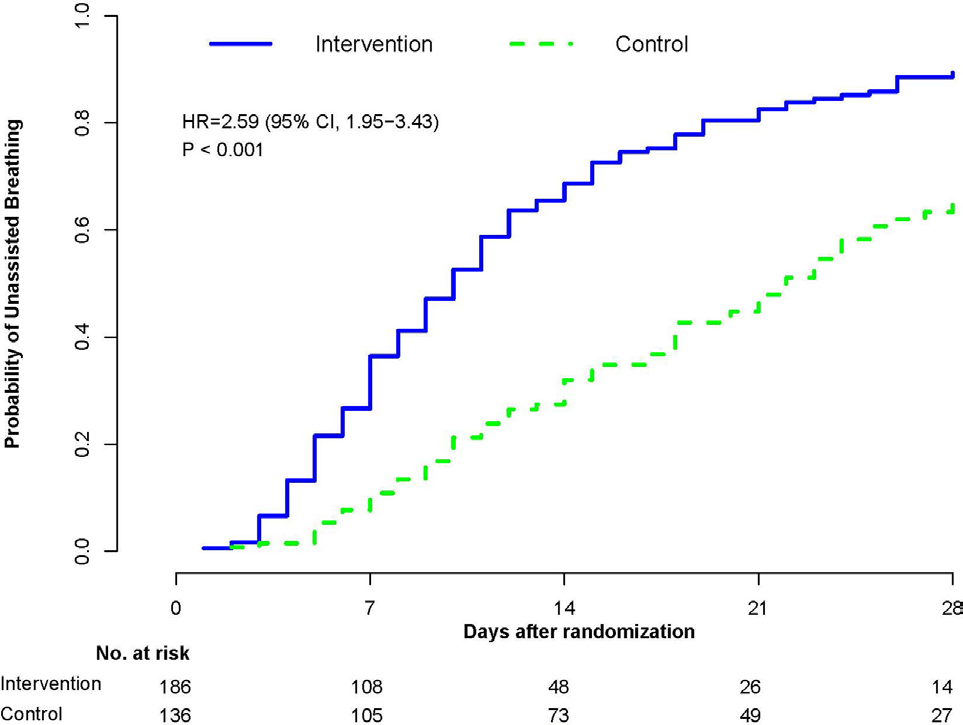
**Probability of achieving unassisted breathing from randomization (methylprednisolone vs. placebo) to hospital discharge or day 28**. Estimated cumulative incidence of achieving (initial) unassisted breathing by day 28 for patients with ARDS (*n* = 322) receiving either prolonged methylprednisolone treatment (blue solid line) or usual care (green dashed line). Death before achieving unassisted breathing is considered a competing risk. By day 28, the methylprednisolone group achieved initial UAB earlier (12.4 ± 0.61 vs. 19.8 ± 0.78 days; HR 2.59, 95% CI 1.95–3.43, *p* < 0.001). Reproduced with permission (58).

Importantly, continuation of methylprednisolone administration after achieving UAB is essential to preserve the significant improvements achieved under treatment. The ARDS network trial (32), contrary to the other RCTs (29–31), had significantly (*p* < 0.001) shorter duration of treatment (Figure 4) as a result of rapid tapering soon after extubation (3.1 ± 1.1 days) (59). “The ARDS network trial (32), in comparison to the other trials (29–31), reported more methylprednisolone patients returning to AB (26 vs. 7%; *p* < 0.008) and to the ICU (21 vs. 2%; *p* = 0.007) by day 28. Return to AB (without reinstitution of glucocorticoid treatment in patients with adrenal suppression) was associated with higher hospital mortality (21 vs. 4%; *p* = 0.003), a factor recognized by the authors (32) to be an important reason why early significant physiological and survival (discharged home after initial wean: 62 vs. 49%, *p* = 0.006) (58) benefits did not translate into a 60-day survival (primary outcome) advantage.”

The ARDS network original report (32) found increased 60-day mortality for methylprednisolone-treated patients randomized after day 13 (32). “This subgroup (*n* = 48), however, had an uncharacteristically low mortality (8%) and large differences in baseline characteristics (60). When the analysis was adjusted for the imbalances at baseline, the mortality difference lost significance (25.6 vs. 13.2%; *p* = 0.325) (61). Irrespective of the interpretation of these data, there is a broad consensus that if glucocorticoid treatment is to be initiated, it should be initiated before day 14 of ARDS (62). For patients randomized before day 14 of ARDS onset, in the methylprednisolone trials, there was a 19% absolute reduction in hospital mortality (20 vs. 39%, *p* < 0.001) and a hazard ratio of 0.51 in time to death by hospital discharge or day 28 (95% CI 0.32–0.83) after adjusting for SOFA score and age. With treatment initiated before day 14, the number needed to treat (NNT) to save one life is 5.3.”

While the observed mortality benefits – originating from small-to-moderate size trials and investigating different treatment protocols – should be accepted with caution, concordance with the trial-level meta-analysis on hospital mortality (29–36) (36 vs. 49%, risk ratio 0.76, 95% CI 0.59–098; *p* = 0.035, *I*^2^ = 17%; moderate certainty) provides increased confidence for a survival benefit for those randomized before day 14 of ARDS. The benefits persist when the analysis includes a newly presented RCT (*n* = 197) (37) (29 vs. 44%; RR 0.62, 95% CI 0.44–0.86, *p* = 0.005; *I*^2^ = 46%). Additional support is also provided by the results of seven cohort studies with historical or concurrent control (*n* = 544) (38–43) reporting a significant mortality reduction (29 vs. 44%; RR 0.68, F-95%, CI 0.57–0.81; *p* < 0.001, *I*^2^ = 46%), and separate meta-analyses showing moderate certainty in a mortality reduction and in prevention of ARDS in patients hospitalized with community-acquired pneumonia (58), and high quality evidence for a mortality reduction for pneumocystis pneumonia (63).

## Glucocorticoid Therapy for Pediatric ARDS

Besides low tidal volume ventilation (64) as part of a “lung protective” ventilation strategy, no other therapeutic approach has consistently shown improvement in the outcomes of children with ARDS or has routinely been implemented in our PICUs (65, 66). Unfortunately, even the effects of low tidal volume ventilation in pediatric ARDS (PARDS) are not based on adequately powered, randomized trials but are extrapolated from adult ARDS studies, single center observational studies, case reports, retrospective chart reviews, and pilot studies (67–70). Similarly, when compared to the adult literature on ARDS, our experience with glucocorticoid therapy for PARDS is limited. Nevertheless, the theoretical indications for using glucocorticoid therapy in PARDS are similar to those of adult ARDS. These include (1) a glucocorticoid receptor-mediated downregulation of systemic and pulmonary inflammation, which is essential for the restoration of tissue homeostasis, disease resolution, and prevention of lung fibrosis (71), and (2) an improvement in capillary-alveolar barrier function, with resolution of alveolar flooding and induction of ENaC (epithelial Na channel) expression and function (72–74). Conversely, concerns about glucocorticoid therapy for children with PARDS revolve around (1) the potential for bone growth retardation or growth arrest (75), (2) suppression of the immature immune system, including a potential increase in nosocomial infections or worsening of an existing infection (76), (3) a potential for glucocorticoid-induced hyperglycemia or systemic hypertension, and (4) psychiatric effects, such as insomnia or delirium (77–79).

Multiple reasons account for our lack of knowledge in regard to glucocorticoid-mediated effects in PARDS, including (1) the incidence of PARDS is lower than of adult ARDS [12.8 vs. 78.9 cases per 100,000 person years (65)], (2) mortality rates from PARDS are lower than from adult ARDS [18 vs. 38.5% (80)], (3) most pediatric intensivists consider glucocorticoid therapy an adjunctive therapy for severe, refractory PARDS, and (4) significant concerns persist among pediatricians regarding the potential adverse effects of glucocorticoids particularly on child growth and the immature immune system. Despite these limitations, it is clearly untenable to administer or withhold glucocorticoids from children with PARDS by simply extrapolating data from adult studies since the disease patterns, immunological responses, capacity for lung growth and development, and clinical outcomes of PARDS differ substantially from those of adult patients (81). PARDS caused by sepsis, pneumonia, and aspiration could conceptually respond favorably to glucocorticoid treatment, but we have little information about the susceptibility or risk factors for PARDS resulting from such triggers.

Several recent studies have confirmed that a large proportion of pediatric intensivists use adjunctive treatments in deteriorating PARDS patients, with glucocorticoids being utilized by 30–35% of pediatricians (82, 83). Interestingly, a Scandinavian study from 2015 reported that 83% of Nordic pediatricians administered glucocorticoids “*sometimes*” for PARDS patients, whereas only 17% admitted to “*never*” using glucocorticoids for PARDS (84).

Martinot et al. published the first report of glucocorticoids administration to children for refractory PARDS in 1997 (85). Six children (age 3–11 years) with moderate–severe PARDS who showed no improvement by days 4–17 of mechanical ventilation were treated with daily methylprednisolone boluses (30 mg/kg) for 3–6 days depending on the observed response. Glucocorticoid treatment was associated with significant improvements in oxygenation between days 1 and 5, and ultimately four of the six children survived. After the initial glucocorticoid bolus, three of the four survivors received a daily methylprednisolone maintenance dose of 2 mg/kg/day followed by a taper for a total treatment duration of 4–11 weeks. The fourth survivor did not receive maintenance therapy after the initial methylprednisolone bolus since a bacterial superinfection was suspected. Interestingly, none of the children sustained any glucocorticoid-related adverse events, including hypertension, nosocomial infections or gastrointestinal bleeding.

Two years later, Goh et al. reported of a 12-month-old with measles-associated refractory PARDS that was successfully treated with a 42-day course of methylprednisolone, consisting of 2 weeks of maintenance therapy and a 2-week taper (86). This protocol was based on the Meduri et al. (31) randomized trial investigating prolonged methylprednisolone treatment in adult patients with unresolving ARDS. However, Goh used significantly higher doses (5 mg/kg bolus, followed by 8 mg/kg/day maintenance) than those used in the Meduri trial (2 mg/kg bolus, 2 mg/kg/day maintenance). Despite the relatively high glucocorticoid dose, the only adverse effect noted was a fungal urinary tract infection, which responded to antifungal therapy and no other infectious, gastrointestinal, or metabolic adverse effects were observed. In 2006, Guglani et al. reported a 21-month-old child with unresolving PARDS related to malnutrition and eosinophilic gastroenteritis successfully treated with a 32-day course of methylprednisolone therapy (87). In contrast to Goh, Guglani administered a lower dose of methylprednisolone (2 mg/kg bolus, followed by 2 mg/kg/day maintenance), similar to the regimen proposed in the 1998 Meduri trial (31). Importantly, this child also suffered no metabolic, cardiovascular, or infectious adverse effects or muscle weakness from glucocorticoid therapy.

Over the past two decades, it became increasingly evident that surfactant loss is a major contributor to the development and progression of adult ARDS and PARDS. Neonatologists have long been aware of the stimulatory effects of glucocorticoids on lung maturation and surfactant production, an idea that is well supported by animal studies. In fact, in young rats with lipopolysaccharide-induced acute lung injury, early administration of dexamethasone (5 mg/kg) increased surfactant protein D levels (88). It seems plausible from this and other studies that glucocorticoids could have similar surfactant-stimulatory effects in PARDS. Although Willson et al. initially found a survival benefit in PARDS patients treated with surfactant (89), this effect was not confirmed in a later study (90). In a surfactant-deficient acute lung injury model, dexamethasone combined with surfactant administration conveyed no additional benefit over surfactant administration alone, despite decreasing nuclear NF-κB levels in lung homogenates (91). The surfactant-stimulating effects of glucocorticoids are well known in neonates, but a link between glucocorticoid administration and improved surfactant levels in PARDS has never been shown. Of interest, the ARDS network reported that patients randomized to the methylprednisolone vs. placebo group had an increased ratio of functional large surfactant aggregates to dysfunctional small aggregates in their cell-free supernatants (28).

While recognizing the difficulties of performing adequately powered RCTs in children, determining the utility of glucocorticoids in PARDS remains a high priority for pediatric intensivists. In 2015, Drago et al. used for the first time a double-blinded RCT design in a pilot study demonstrating the feasibility of administering prolonged, low-dose glucocorticoids and measuring clinically relevant outcomes in children with early PARDS (59). In the 35 patients randomized to placebo or glucocorticoid groups, no differences occurred in duration of mechanical ventilation, ICU stay, hospital stay, or mortality. However, the glucocorticoid group had higher PaO_2_/FiO_2_ ratios on days 8 and 9 and lower CO_2_ levels on days 2 and 3 of treatment, required fewer racemic epinephrine treatments for post-extubation stridor and less supplemental oxygen at PICU discharge. Importantly, glucocorticoid treatment (a single 2 mg/kg IV methylprednisolone bolus, followed by 7 days of 1 mg/kg/day methylprednisolone maintenance therapy and a 7-day taper) was not associated with any adverse effects, such as hyperglycemia or nosocomial infections. The strengths of this study included the enrollment of a generalizable PICU population, a randomized placebo-controlled study design, masked study drug infusions, a standardized regimen of low-dose methylprednisolone, *a priori* definition of primary and secondary outcomes and of potential adverse drug effects, as well as an intention-to-treat analysis with complete follow-up of all enrolled subjects. Limitations of this study included the relatively small sample size (17 and 18 patients in the glucocorticoid and placebo groups, respectively) and failure to strictly standardize ventilator management and ventilator weaning strategies. Although a 2-week glucocorticoid course can be considered as “prolonged” treatment and has been used in several adult ARDS trials, the most prominent benefits in the adult studies occurred with a 4-week course of glucocorticoids (58). Furthermore, although this exploratory study demonstrated the feasibility of prolonged glucocorticoid infusions in PARDS patients without any obvious adverse effects, a proper safety analysis could not be performed with this sample size.

Later in 2015, the same authors published a follow-up study describing alterations in plasma biomarkers in PARDS patients and associations of these biomarkers with the changes in clinical outcomes observed earlier (92). Interestingly, the placebo group showed higher IL-15 and basophil levels at study entry, whereas on day 7 this group showed lower IL-1α, IFN-γ, and IL-10 levels. In contrast, the glucocorticoid group showed lower INF-α, IL-6, IL-10, MCP-1, G-CSF, and GM-CSF levels at study entry, but higher IL-17α levels on day 7. Total and differential cell counts remained unchanged within the placebo group between days 0 and 7, whereas in the glucocorticoid group, total WBC and platelets counts were increased on day 7 (92). The authors also proposed a series of positive and negative correlations between cytokine levels, cell counts, coagulation parameters, and clinical parameters of disease severity. Although inflammatory biomarkers can be successfully measured in PARDS patients with commercially available techniques, limitations of this study include again the small sample size (35 patients), the relatively short study period of 7 days, the inability to compare plasma to BAL samples (an intrinsic problem for most pediatric studies of lung disease), and the lack of functional assays to uncover any mechanistic consequences of these alterations in biomarkers.

In the same year, Yehya et al. published a prospective, observational single center study of 283 PARDS patients describing the association between glucocorticoid exposure for >24 h and the clinical outcomes of these patients (93). Interestingly, similar to the Scandinavian survey, the majority (60%) of PARDS patients in this US-based study received glucocorticoids for >24 h and only 23% did not receive any type of glucocorticoid. However, the glucocorticoid most commonly used was hydrocortisone and not methylprednisolone, the most commonly administered glucocorticoid in large-scale, adult ARDS trials showing improvements in outcomes. Yehya et al. found that all-type glucocorticoid exposure for >24 h was associated with increased mortality in PARDS, fewer ventilator-free days at 28 days, and longer duration of mechanical ventilation in survivors, although after multivariate and propensity score adjustments, the increase in mortality was no longer detectable. Strengths of this study include the larger sample size and meticulous documentation of all types of glucocorticoid exposures. The study also revealed that currently pediatricians use multiple types of glucocorticoids for PARDS patients and for a variety of indications, with shock ranking as the most common. However, the outcomes of this study stand in marked contradiction to multiple, large-scale, and highly powered, randomized adult ARDS trials, which consistently show improvements in clinical outcomes and inflammatory biomarkers. This discrepancy may be explained by several limitations of the Yehya study, including (1) not recording the specific indication for glucocorticoid administration, (2) grouping unexposed and short-term (<24 h) glucocorticoid-exposed patients together as a control group, and (3) no implementation of a glucocorticoid weaning protocol to prevent rebound inflammation, which is a key feature of the adult, glucocorticoid-favoring ARDS trials. These flaws create major challenges in the interpretation of the data and in assigning clinical relevance to these findings.

Clearly, much more research is needed before we can make an educated decision about the use of glucocorticoids for all, or even some, PARDS patients. Nevertheless, most recently the expert panel of the 2015 Pediatric Acute Lung Injury Consensus Conference recommended with “strong agreement” against the use of glucocorticoid therapy for PARDS. These experts highlighted the need for future research to identify specific pediatric patient populations most likely to benefit from glucocorticoid therapy and encouraged the development of pediatric dosing and delivery regimens (94). Another expert review proposed that ARDS patients with adrenocortical insufficiency would demonstrate greater benefit with glucocorticoids as compared to patients with an intact hypothalamic–pituitary–adrenal axis (95).

## Future Research Priorities

1. The relatively low incidence and mortality rates from PARDS in comparison to adult ARDS mandate the careful design of a large, multicenter RCT to determine the effects of prolonged glucocorticoid therapy on PARDS outcomes, such as mortality, duration of mechanical ventilation, ICU-free days, or hospital stay, since a single center study is unlikely to achieve adequate power. Drago et al. calculated that in order to achieve a 15, 28, or 36% reduction in the duration of mechanical ventilation, we need to enroll 547, 137, or 98 subjects, respectively, in the placebo and the treatment groups (assuming an α-error of 0.01 and a power of 80%) (59). The design of this study should aim at standardizing not only the glucocorticoid protocol but also the mechanical ventilation and ventilator weaning protocols, as well as sedation, fluid management, and nutrition strategies.
2. Although it is safe to assume that patients with different PARDS etiologies will respond differently to prolonged glucocorticoid therapy, an initial trial should enroll all patients who meet PARDS criteria based on the 2015 Pediatric Acute Lung Injury Consensus Conference (94). Stratification of enrollment according to their PARDS etiologies will allow a balanced distribution of PARDS etiologies in the treated and untreated groups, as well as subgroup comparisons of glucocorticoid effectiveness for different subpopulations within these strata.
3. Efforts to define the side effects and safety profile of prolonged, low-dose glucocorticoid administration should receive a high priority. While Drago et al. showed the feasibility of administering prolonged glucocorticoid infusions to children with PARDS using a randomized, double-blinded study design, due to the relatively low number of patients enrolled, a safety analysis could not be performed, which needs to be assessed in futures studies.
4. The molecular and clinical consequences of potential rebound effects after glucocorticoid discontinuation are unknown in children with PARDS. In the adult ARDS trials, a prolonged glucocorticoid weaning protocol was required to sustain improved outcomes. In contrast, rapid discontinuation of glucocorticoids, including in the ARDSnet trial, consistently led to increased readmission rates to the ICU, reinstitution of mechanical ventilation, and increased mortality (31, 96). It is unknown whether clinical deterioration after abrupt glucocorticoid discontinuation holds true for children with PARDS.
5. In both adult and pediatric ARDS trials, prolonged glucocorticoid treatment was not associated with increased risk of nosocomial infections. Other glucocorticoid-induced adverse effects, such as hyperglycemia and hypertension, are rarely reported and easily controlled. Nevertheless, the effects of prolonged glucocorticoid therapy on the immune response of chronically immunosuppressed children (such as solid organ and bone marrow recipients) or children with autoimmune diseases remain unknown. In addition, we need to determine the effects of glucocorticoid therapy for PARDS on children who are chronically exposed to glucocorticoids but not immunosuppressed in the classical sense [e.g., inhaled glucocorticoids for long-term asthma or cystic fibrosis control, or nasal glucocorticoids for allergic rhinitis (97)].
6. Many pediatricians are concerned about the long-term consequences of prolonged glucocorticoid exposure on bone growth and child development. The widespread use of short-term glucocorticoid bursts (e.g., for status asthmaticus or acute adrenal crisis) does not appear to affect long-term growth. On the other hand, exposure to inhaled or oral glucocorticoids for months to years (e.g., asthma control therapy) may decrease the growth potential of children (79). Significant negative effects on bone growth after glucocorticoid exposure for 2 weeks, as proposed in the study design by Drago et al. (59), is unlikely to affect the final height of children, but this needs to confirmed. In contrast, the adult ARDS trials recommend glucocorticoid therapy for 4 weeks (96), potentially carrying greater risk for growth-related long-term effects. Interestingly, critically ill children who suffered burn wounds actually show an increase in lean body mass, bone mineral content, and bone mineral density after prolonged exposure to synthetic glucocorticoids (98). These findings should encourage us to consider different types of glucocorticoids for PARDS therapy in order to minimize potential adverse effects.
7. The consequences of a prolonged glucocorticoid course on the effectiveness of routine vaccinations and vaccination catch-up schedules are unknown and need to be determined.
8. Dysregulated acute and chronic systemic inflammation is associated with long-term systemic morbidity in ARDS survivors, including muscle weakness with impaired physical function (99) and neurocognitive impairment (100). PARDS RCTs must include long-term follow-up evaluating functional status and cognitive function.

In summary, although a robust body of adult literature points toward improved outcomes of ARDS patients treated with prolonged glucocorticoid regimens, including a slow, standardized glucocorticoid wean, the relative lack of pediatric data should encourage us to maintain equipoise toward glucocorticoid therapy for PARDS. We should neither assume that the adult results can be directly applied to a pediatric population, nor should we propagate generalized statements that all exposures to glucocorticoids, including a 4-week treatment course for PARDS, will increase the risk of nosocomial infections, cause hypertension, hyperglycemia, or growth retardation, since this is neither supported by the adult nor pediatric literature on glucocorticoid use in ARDS. Clearly, there is an urgent need for large-scale, well-designed RCTs in Pediatrics to determine the role of prolonged glucocorticoid therapy for PARDS.

## Author Contributions

All authors listed, have made substantial, direct and intellectual contribution to the work, and approved it for publication.

## Conflict of Interest Statement

The authors declare that the research was conducted in the absence of any commercial or financial relationships that could be construed as a potential conflict of interest.
